# Evaluation of large language models as a diagnostic aid for complex medical cases

**DOI:** 10.3389/fmed.2024.1380148

**Published:** 2024-06-20

**Authors:** Alejandro Ríos-Hoyo, Naing Lin Shan, Anran Li, Alexander T. Pearson, Lajos Pusztai, Frederick M. Howard

**Affiliations:** ^1^Yale Cancer Center, Yale School of Medicine, New Haven, CT, United States; ^2^Department of Medicine, University of Chicago, Chicago, IL, United States

**Keywords:** large language model (LLM), ChatGPT, complex clinical cases, diagnosis, clinical case solving

## Abstract

**Background:**

The use of large language models (LLM) has recently gained popularity in diverse areas, including answering questions posted by patients as well as medical professionals.

**Objective:**

To evaluate the performance and limitations of LLMs in providing the correct diagnosis for a complex clinical case.

**Design:**

Seventy-five consecutive clinical cases were selected from the Massachusetts General Hospital Case Records, and differential diagnoses were generated by OpenAI’s GPT3.5 and 4 models.

**Results:**

The mean number of diagnoses provided by the Massachusetts General Hospital case discussants was 16.77, by GPT3.5 30 and by GPT4 15.45 (*p* < 0.0001). GPT4 was more frequently able to list the correct diagnosis as first (22% versus 20% with GPT3.5, *p* = 0.86), provide the correct diagnosis among the top three generated diagnoses (42% versus 24%, *p* = 0.075). GPT4 was better at providing the correct diagnosis, when the different diagnoses were classified into groups according to the medical specialty and include the correct diagnosis at any point in the differential list (68% versus 48%, *p* = 0.0063). GPT4 provided a differential list that was more similar to the list provided by the case discussants than GPT3.5 (Jaccard Similarity Index 0.22 versus 0.12, *p* = 0.001). Inclusion of the correct diagnosis in the generated differential was correlated with PubMed articles matching the diagnosis (OR 1.40, 95% CI 1.25–1.56 for GPT3.5, OR 1.25, 95% CI 1.13–1.40 for GPT4), but not with disease incidence.

**Conclusions and relevance:**

The GPT4 model was able to generate a differential diagnosis list with the correct diagnosis in approximately two thirds of cases, but the most likely diagnosis was often incorrect for both models. In its current state, this tool can at most be used as an aid to expand on potential diagnostic considerations for a case, and future LLMs should be trained which account for the discrepancy between disease incidence and availability in the literature.

## 1 Introduction

Large language models (LLMs) are complex, neural network-based models trained on vast amounts of text to accurately interpret human language. LLMs have been applied to a wide range of tasks within medical science, including simplifying radiology reports, accurately responding to questions posted by patients on an internet forum, generating realistic medical abstracts, and predicting in-hospital mortality (1–4). Although LLMs have shown passable accuracy in answering medical licensing exam questions in numerous studies (1–5), it is unclear if this performance can be leveraged to serve as a decision aid in real clinical practice, where cases have nuance beyond that of standardized testing. Given the widespread uptake of LLMs, they have been proposed as a diagnostic decision aid for students, and are likely in use despite the limited knowledge about specific model performance (6). Chat GPT (Generative Pre-trained Transformer) is a natural language processing model that became publicly available in November 2022, it provides outputs in response to inputs or prompts, learning its skills from internet data.

Different versions of GPT are currently available, GPT3.5 is a Chatbot based on the GPT3.5 model, whereas the GPT4 foundation features an approximately 1,000-fold increase in model parameters and an expanded context window length, resulting in an enhanced capability of solving complex tasks (7–9). GPT can be used to write computer code, analyze text, draft documents, create conversational agents, and has been shown to proficiently answer different standardized tests (7, 10) it has a considerable semantic medical knowledge and has been shown to be capable of medical reasoning (10). This has been reflected by its capabilities in answering medical questions (11), simplifying radiology reports, performing well at medical licensing exams, among others (1–4). It is currently considered an attractive tool in diverse settings of medicine, however these LLMs could potentially contribute to misinformation and exacerbate scientific misconduct in the setting of a lack of accountability and transparency.

This study aimed to characterize the performance and consistency of LLMs in diagnosing a series of challenging case records published from a single institution. In this study, we evaluated OpenAI’s GPT-3.5 and GPT-4 models to establish a baseline for models trained on general (as opposed to medical-specific literature), as well as to identify patterns in misdiagnosis to inform fine-tuning of diagnostic decision aids. In this study we used cases from the Massachusetts General Hospital Case Records which have been published since 1923 in the New England Journal of Medicine. These cases have been used as teaching tools illustrating different clinical cases, and the workup of the differential diagnosis of frequently uncommon diseases or uncommon disease presentations (12). We introduced the case presentation of these clinical cases and asked GPT to provide a list of the most likely differential diagnosis.

## 2 Methods

Seventy-five sequential clinical cases were retrieved from the case records of the Massachusetts General Hospital, published in the New England Journal of Medicine, from January 2022 to November 2023 (12). This period was selected to ensure cases did not overlap with the training data for the LLMs. The case presentation was truncated prior to the discussant’s review of the differential diagnosis, and text referencing figures or tables was removed. A uniform prompt requesting a differential diagnosis for the case presentation text was provided to OpenAI’s GPT-3.5 (gpt-3.5-turbo) and GPT-4 (gpt-4) models. First, three prompts were tested on a subset of 10 cases for four replicates each. The prompts included (1) ‘*please read the following case, and provide a differential diagnosis for the underlying cause of this presentation*’; (2) as per (1) with the modification ‘…*provide a thorough and specific list of differential diagnosis…*’; and (3) as per (2) with the additional sentence *‘please list the diagnosis that most explains all the features of the presentation first, and include rare diagnoses if they are the best explanation for the presentation*.’ All prompts yielded similar lists, but the prompt (3) yielded diagnosis lists that most frequently listed the correct diagnosis first, and was chosen for all subsequent analysis. All clinical cases were queried with this prompt, with four replicates performed for each model (Supplementary Table 1).

The rank order of the correct diagnosis within the differential diagnosis list was established by consensus of study authors. The overlap between the full list of differential diagnoses provided by GPT and by the case discussant was similarly compared. Finally, accuracy of LLMs was correlated with disease incidence (estimated from literature review of PubMed as well as cdc.gov with references listed in Supplementary Table 1, as indexed by Google both with the search term ‘diagnosis’ incidence), with rare diseases without estimable incidence such as those only described in case reports assigned an incidence of 0.1 per 100,000, as well as representation of the diagnosis in medical literature as assessed by article count returned when searching for the diagnosis (or simplified surrogate term, as listed in Supplementary Table 1) in PubMed (conducted with an article cutoff of April 21st, 2023).

### 2.1 Statistical analysis

A Mann–Whitney U test was used to compare the number of diagnoses provided by case discussants and GPT models. A Fisher’s exact test was used to compare whether the first diagnosis was the correct diagnosis, whether among the top three diagnosis was the correct diagnosis, whether the correct diagnosis was in the list of differential diagnosis from GPT3.5 and 4. To assess whether GPT was able to provide the correct diagnosis among different medical specialties, five groups were designated [Group 1: neurology and psychiatry; group 2: oncology and hematology; group 3: infectious diseases, internal medicine, endocrinology and toxicology; group 4 rheumatology, allergy and autoimmune diseases; group 5: others (cardiology, gastroenterology, genetic diseases, dermatology, nephrology and pediatrics)], A Fisher’s exact test was used to compare results between GPT 3.5 and 4. A multivariable logistic regression model was used to determine the association between disease incidence and PubMed article count with these same three performance metrics. To assess the similarity between the differential diagnosis lists, the Jaccard similarity index was used (ranging from 0 to 1, 0 reflects no similarity, whereas 1 reflects a complete similarity between the analyzed sets), utilizing each case entry repeat, to test differences between GPT 3.5 and 4, a Mann–Whitney test was performed. To assess reproducibility across iterations of each model, intraclass correlation coefficients (ICC) were calculated using the two-way mixed effects, absolute agreement, multiple raters/measurements formulation (13), values of <0.5 and > 0.9 reflect poor and excellent reliability, respectively. Statistical analyses and graphs were performed using GraphPad Prism 9.0 (GraphPad Software, Inc., San Diego, CA) and Python version 3.7.5 (Python Software Foundation) using statsmodels 0.13.2.

## 3 Results

### 3.1 Accuracy of GPT models in complex diagnostic challenges

**Figure 1:**
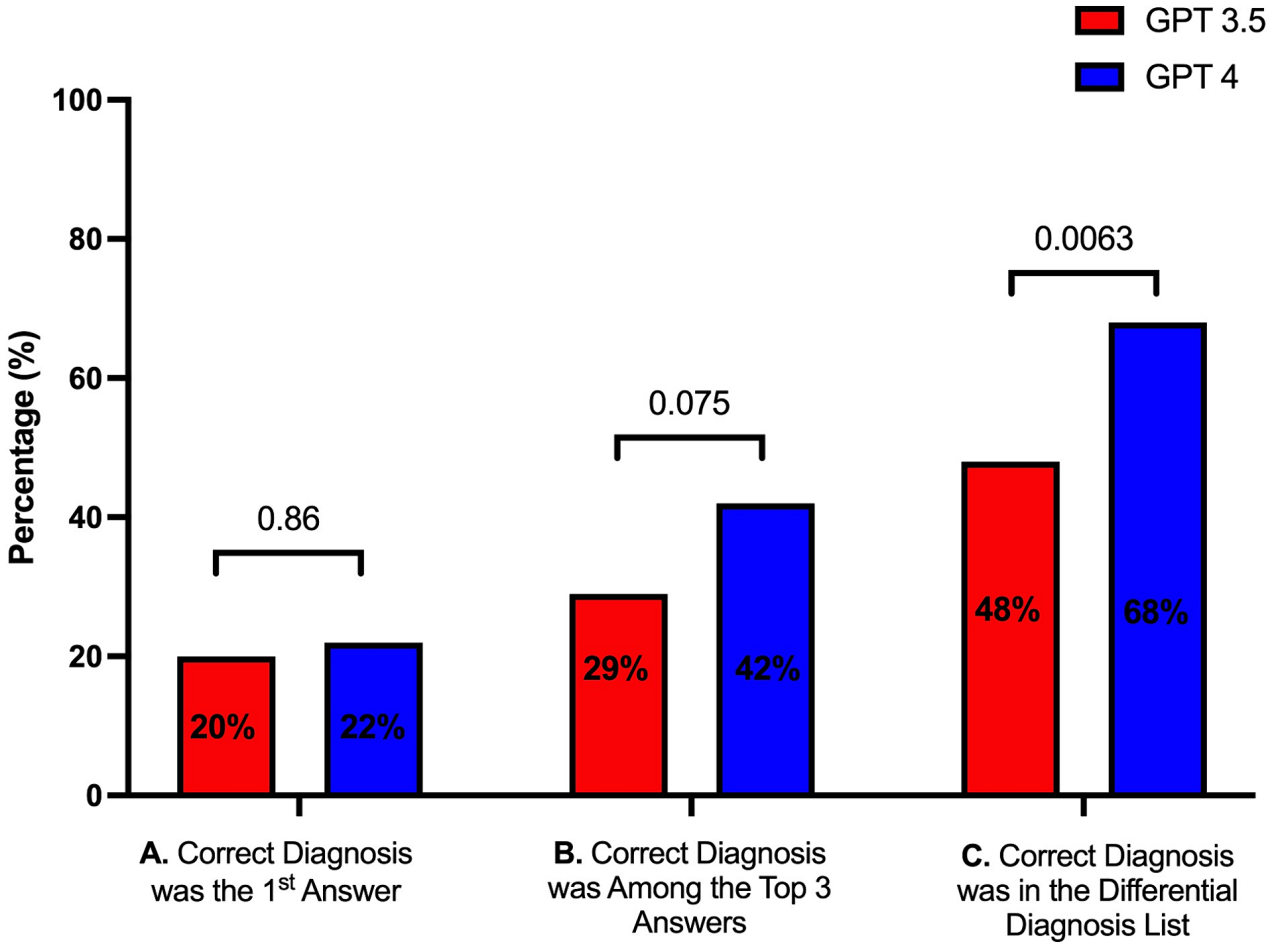
Performance of GPT3.5 and GPT4 in providing **(A)** the first diagnosis as the correct diagnosis, **(B)** the correct diagnosis among the top three diagnoses, and **(C)** the correct diagnosis among the entire list of diagnoses.

Seventy-five cases from the Massachusetts General Hospital Case Records were introduced to the two GPT models. Compared to the case discussants, who provided a mean of 16.77 [interquartile range (IQR) (representing the distance between the first and the third quartile) 12] diagnoses, GPT4 produced a similar number (mean 15.45, IQR 11, *p* = 0.302) of unique diagnoses over four replicates, whereas GPT3.5 listed significantly more diagnoses (mean 30, IQR 10, *p* = <0.0001). GPT4 included the correct diagnosis in its differential list in two thirds (68%) of cases, with the correct diagnosis included in the top 3 items in the differential in 42% of cases, in contrast GPT3.5 included the correct diagnosis in its differential list in half (48%, *p* = 0.006) of the cases, and the correct diagnosis included in the top three differential diagnoses in 29% (*p* = 0.075) of the cases, thus observing that GPT4 outperforming GPT3.5 in both metrics (Figure 1). GPT4 was able to formulate more specific answers that better depicted the true diagnosis in many cases. For example, in Case 6–2022 (Immune checkpoint inhibitor-induced diabetes), GPT3.5 was only able to vaguely link the presentation to immunotherapy - “Side effects of cancer treatment: The patient’s symptoms could be side effects of cancer treatment such as pembrolizumab…” - whereas GPT4 concisely answered “Pembrolizumab-induced diabetes mellitus.”

### 3.2 Consistency of GPT model diagnostic lists

**Figure 2:**
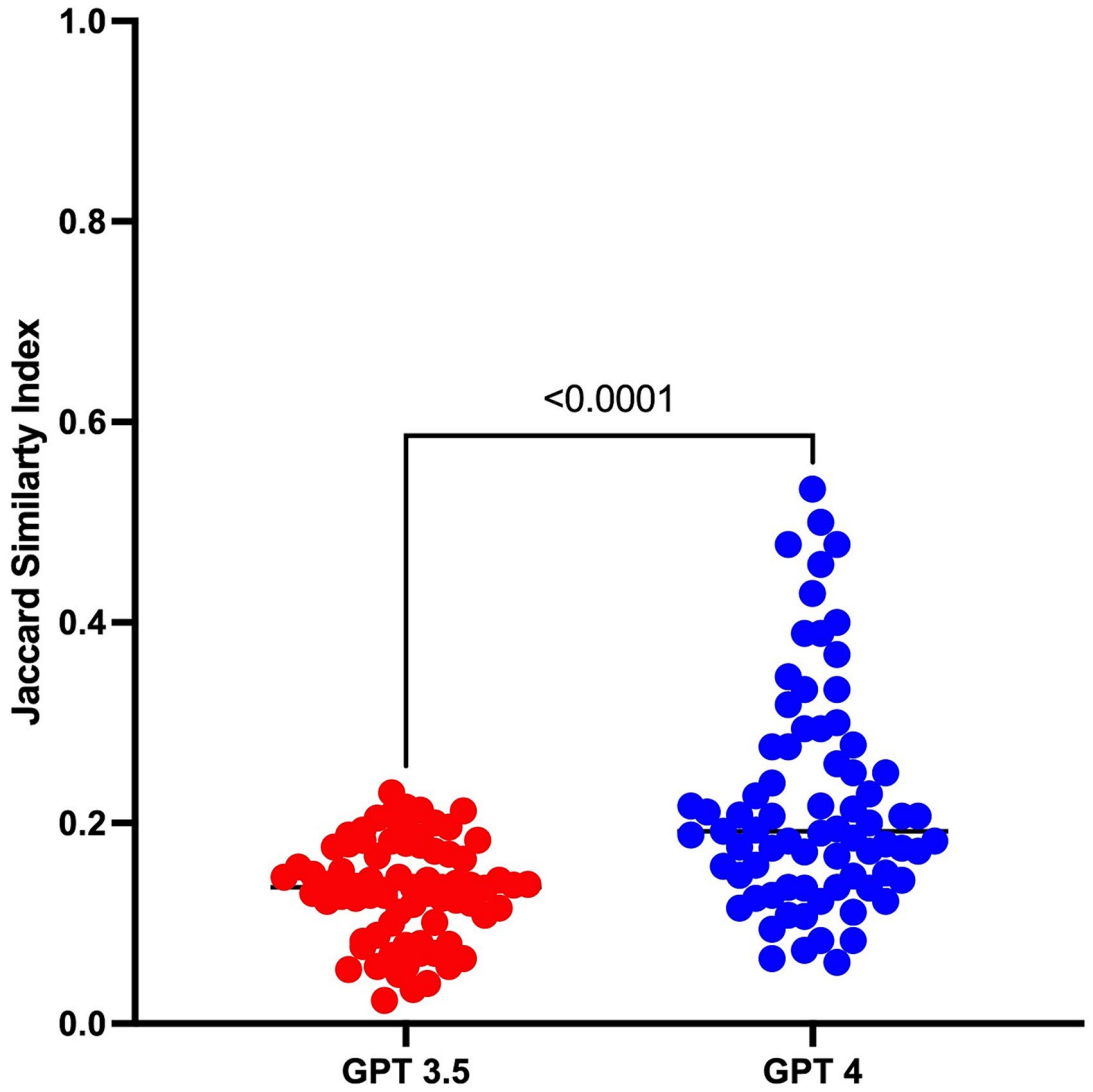
Jaccard Similarity Index indicating the overlap between GPT3.53/GPT4 and the differential provided by the case discussant.

As the results of GPT models may differ across repetitions, it is important to understand how the prioritization of diagnoses might change if these tools are clinically implemented. Ranking of the correct diagnosis within a differential was more consistent across repetitions for GPT4 (ICC 0.65, 95% CI 0.42–0.80) than with GPT3.5 (ICC 0.37, 95% CI–0.25 – 0.71). The differential diagnosis list generated by GPT4 also had greater overlap with the discussant’s list (Jaccard Similarity Index 0.22, IQR 0.12) than GPT3.5 (0.13, IQR 0.076, *p* = <0.0001, Figure 2) – although overlap was fair at best.

### 3.3 Associations of model accuracy with medical specialty and disease incidence

Each case was classified into medical specialties groups (*n* = 5), among these groups, GPT4 was numerically and statistically superior to GPT3.5 in all categories except in the Rheumatology, Allergy, and Autoimmune Diseases category (Table 1). We also assessed whether model accuracy was dependent on disease incidence or representation in the literature. PubMed article count for the correct diagnosis was associated with a greater likelihood that the diagnosis would be included in the differential generated by GPT3.5 (Odds Ratio (OR) 1.40, 95% CI 1.25–1.56, *p* < 0.001) and GPT4 (OR 1.25, 95% CI 1.13–1.40, *p* < 0.001). Similar trends were seen for likelihood of a diagnosis being listed first or within the top 3 generated diagnoses (Table 2). Conversely, disease incidence was either a neutral or negative effect on the likelihood of a diagnosis being listed by either model.

**Table 1:** Performance of GPT 3.5 and 4 in providing the correct diagnosis, according to medical specialty.

	GPT 3.5 (%)	GPT 4 (%)	OR (95% CI)	*p*-value
Group 1 (*n* = 9)	41	72	5.2 (1.94–14.23)	0.0019
Group 2 (*n* = 24)	60	83	5.6 (2.95–10.73)	<0.0001
Group 3 (*n* = 19)	23	53	4.92 (2.39–9.77)	<0.0001
Group 4 (*n* = 13)	64	60	1.36 (0.62–3.04)	0.55
Group 5 (*n* = 10)	50	65	2.78 (1.10–6.86)	0.043

Odds ratios [OR] comparing GPT 4 vs. 3.5. Group 1: Neurology and Psychiatry, Group 2: Oncology and Hematology, Group 3: Infectious Diseases, Internal Medicine, Toxicology, Group 4: Rheumatology, Autoimmune Diseases, Group 5: Others (Cardiology, Genetic Diseases, Gastroenterology, Dermatology, Nephrology and Pediatrics).

**Table 2:** Performance of GPT 3.5 and 4 in providing the correct diagnosis, according to disease incidence and PubMed articles covering the disease.

	Top diagnosis correct		Correct diagnosis in top 3		Correct diagnosis in differential	
	OR (95% CI)	*p*-value	OR (95% CI)	*p*-value	OR (95% CI)	*p*-value
GPT 3.5						
Incidence (per 10-fold increase)	0.80 (0.67–0.95)	0.01	0.74 (0.64–0.87)	< 0.001	0.82 (0.74–0.92)	< 0.001
PubMed Articles (per 10-fold increase)	1.32 (1.12–1.56)	0.001	1.42 (1.23–1.64)	< 0.001	1.40 (1.25–1.56)	< 0.001
GPT 4						
Incidence (per 10-fold increase)	0.90 (0.80–1.02)	0.108	0.90 (0.81–0.99)	0.036	0.90 (0.82–0.99)	0.033
PubMed Articles (per 10-fold increase)	1.15 (1.01–1.30)	0.03	1.16 (1.04–1.28)	0.005	1.26 (1.13–1.40)	< 0.001

Odds ratios [OR] listed for a multivariate logistic regression including both incidence and article count.

## 4 Discussion

We have demonstrated here a comprehensive characterization of the accuracy and reproducibility of two GPT models in solving complex clinical case scenarios. Whereas high accuracy was seen when evaluating GPT-3 in diagnosing common presentations such as upper respiratory tract infections (14), we found that in approximately one third of cases the best model failed to identify the correct diagnosis in complex cases. Thus, although current GPT models are insufficient to replace physician expertise, they may have some clinical utility as a diagnostic checklist (15) to reduce error when physicians are presented with a puzzling clinical scenario.

It is worth noting that although GPT3.5 was able to provide a longer list of differential diagnoses, these did not present a better concordance with the Massachusetts General Hospital case discussants diagnoses. Furthermore, GPT4 was not only better at providing the first diagnosis as the correct diagnosis, but it outperformed GPT3.5 in providing the correct diagnosis among the differential diagnosis lists.

A similar study by Zahir and collogues (16) used GPT and cases from the Massachusetts General Hospital case records to assess whether the model’s diagnoses matched the final case diagnosis, their results found an agreement between GPT4’s top diagnosis and the final diagnosis in 39% of the cases, and in 64% of the cases the final diagnosis was included in the differential diagnosis list. These results contrast with ours, since we found that GPT4 was able to provide the correct diagnosis as the first answer in 22% of the cases, whereas it provided the correct diagnosis within the differential diagnosis list in 68% of the cases. In addition, Zhair’s study found that GPT4 provided a mean of 9 differential diagnoses, similarly our study found a mean of 9.23 diagnoses.

Another study using a different, medicine-specific large language model called Med-PaLM, was able to provide accurate answers to different questions posted in a multiple-choice and long-form setting. Med-PaLM was superior in solving medical questions when compared to MultiMedQA (6 sets of open data that include similar questions to the United States Medical Licensing Examination (USMLE)), and HealthSearchQA (related to common consumer health related questions). MedPaLM was able to answer accurately different formats of questions, such as multiple choice and long form. In a second part of the study, clinicians from different countries were asked to solve 140 medical questions in long-form answers, the same task was performed by MedPaLM. The answers were assessed by clinicians with specialties in different medical fields, the answers provided by the LLM overall presented outstanding results, however MedPaLM’s answers presented higher numbers of incorrect information, which most of the times was clinically significant (11).

When formulating a differential diagnosis, disease incidence as well as the severity/consequences of missed diagnosis are often considered (17). However, some common diseases are underrepresented in the literature, whereas some rare conditions are given particular emphasis in medical literature and educational materials. In an attempt to refine medical-domain performance, several models have been trained specifically on PubMed, which may be subject to this same bias (18). As LLMs are refined as diagnostic decision aids, strategies to align output with true disease prevalence are needed.

## 5 Limitations

One of the limitations of this study was the lack of publicly available diagnostic challenges with curated differential diagnosis lists, resulting in our use of a single source of cases which was only modest in size. The small sample size may lead to lower accuracy in precisely quantifying the difference in performance between the GPT models tested. Additionally, the Massachusetts General Hospital Case Records present complex cases that may not represent the most frequent case presentations – which may be more straightforward with higher diagnostic accuracy from AI models.

As the GPT models evaluated were trained on data collected on or before September 2021, and thus performance for certain diagnoses with changing epidemiology [such as monkeypox (19)] may be underestimated. We chose to evaluate OpenAI’s GPT models in this study rather other LLMs due to their widespread uptake (20), as it is most likely to be in current use by physicians and trainees, and as such characterization of performance is most urgent. Furthermore, we used a single prompt to evaluate model performance in our primary analysis. Although preliminary analysis suggested that performance was similar across prompts, it is possible that modifications of the prompt may change the relative accuracy of GPT3.5 and 4 models.

Finally, although we found that disease incidence was either not associated or negatively associated with model accuracy, incidence is difficult to establish and these estimates represent our best efforts to define incidence through literature review. Incidence can vary widely depending on the population studied and across geographic regions, and these results may differ with alternate approaches to estimate incidence.

## 6 Conclusion

In this study we demonstrated that OpenAI’s GPT-4 model outperformed GPT-3.5 in correctly diagnosing challenging clinical cases, but misdiagnosis was common, and at best such models might be used as decision aids in their current state. In training LLMs specifically as diagnostic aids, steps should be taken to account for the overrepresentation of some diagnoses in the medical literature. It is important to take into consideration certain aspect of using LLM in medicine, such as a negative impact in critical thinking, ethical considerations, as well as potentially detrimental consequences for the patient, thus the use of LLM in clinical medicine might not be ready for a global integration into clinical workflows.

## Data availability statement

The original contributions presented in the study are included in the article/Supplementary material, further inquiries can be directed to the corresponding authors.

## Author contributions

AR-H: Writing – review & editing, Writing – original draft, Visualization, Validation, Supervision, Software, Project administration, Methodology, Investigation, Formal analysis, Data curation, Conceptualization. NS: Writing – review & editing, Methodology, Investigation, Formal analysis, Data curation. AL: Writing – review & editing, Software, Methodology, Investigation, Formal analysis. AP: Writing – review & editing, Software, Methodology, Investigation, Formal analysis, Data curation. LP: Writing – review & editing, Visualization, Validation, Supervision, Software, Resources, Project administration, Methodology, Investigation, Funding acquisition, Formal analysis, Data curation, Conceptualization. FH: Writing – review & editing, Writing – original draft, Visualization, Validation, Supervision, Software, Resources, Project administration, Methodology, Investigation, Funding acquisition, Formal analysis, Data curation, Conceptualization.
